# Malignant ventricular tachycardia in acromegaly: a case report: Taquicardia ventricular maligna na acromegalia: relato de caso

**DOI:** 10.1590/1516-3180.2012.6410005

**Published:** 2014-07-19

**Authors:** Zhe An, Yu-quan He, Guo-hui Liu, Li-li Ge, Wen-qi Zhang

**Affiliations:** I MD. Specialist in Cardiology, Department of Cardiology, China-Japan United Hospital of Jilin University, Changchun, China.; II MD. Department of Cardiology, China-Japan United Hospital of Jilin University, Changchun, China.; III MD. General Physician, Department of Cardiology, China-Japan United Hospital of Jilin University, Changchun, China.; IV MD, PhD. Professor, Department of Cardiology, China-Japan United Hospital of Jilin University, Changchun, China.

**Keywords:** Tachycardia, ventricular, Acromegaly, Case report [publication type], Defibrillators, implantable, Arrhythmias, cardiac

## Abstract

**CONTEXT:**

In patients with acromegaly, cardiovascular complications are the main cause of death; sudden death has been associated with ventricular tachyarrhythmias. In other patients with life-threatening malignant ventricular tachyarrhythmias, surgical placement of an implantable cardioverter-defibrillator (ICD) has proved highly effective in reducing sudden death rates.

**CASE REPORT:**

The present article reports the case of a 50-year-old male acromegalic patient who presented symptoms of syncope induced by ventricular tachycardia. An ICD was surgically implanted and a pituitary adenoma, which was responsible for the acromegaly, was completely removed in the same procedure. The surgery was successful and the ventricular arrhythmias were effectively terminated. During six months of follow-up, no documented arrhythmic episodes occurred.

**CONCLUSION:**

In patients with acromegaly, malignant ventricular tachyarrhythmia might be effectively controlled by implantation of an ICD and surgical removal of the pituitary adenoma.

## RESUMO

**CONTEXTO:**

As complicações cardiovasculares são a principal causa de morte em pacientes com acromegalia, e a morte súbita tem sido associada a taquiarritmias ventriculares. Em outros pacientes com risco de vida por taquiarritmias ventriculares malignas, a aplicação cirúrgica de um cardioversor-desfibrilador implantável (CDI) provou ser altamente eficaz na redução das taxas de morte súbita.

**RELATO DE CASO:**

O presente artigo relata o caso de um paciente acromegálico de 50 anos de idade e do sexo masculino, que apresentava sintomas de síncope induzida por taquicardia ventricular. Foi implantado cirurgicamente nesse paciente um CDI e na mesma intervenção cirúrgica foi completamente removido um adenoma hipofisário responsável pela acromegalia. A cirurgia foi bem-sucedida e o paciente deixou de sofrer de arritmias ventriculares. Durante seis meses de acompanhamento, não se documentaram, nesse paciente, episódios arrítmicos.

**CONCLUSÃO:**

A taquiarritmia ventricular maligna pode ser efetivamente controlada em pacientes com acromegalia pela implantação de um CDI combinado com a remoção cirúrgica do adenoma hipofisário.

## INTRODUCTION

Acromegaly is characterized by hypersecretion of growth hormone, which in more than 95% of patients is caused by a secreting pituitary tumor.^1^ The high prevalence of arrhythmias and sudden cardiac death in these patients may be related to myocardial interstitial fibrosis.^2^ Several clinical studies have reported the presence of premature ventricular contractions,^3^ third-degree atrioventricular block^4^^,^^5^ and dilated cardiomyopathy^6^ in patients with acromegaly. However, in a review of the literature regarding case reports on malignant ventricular tachyarrhythmia concomitant with hemodynamic instability and acromegaly, only one case was found. In 2010, Yokota et al. reported that a 51-year-old man with a diagnosis of cardiac dysfunction with acromegaly was successfully treated by means of transsphenoidal surgery and normalization of growth hormone and insulin-like growth factor-1 (IGF-1), even in the presence of severe fibrosis in the myocardium.^7^ In the present report, we describe a case of an acromegalic patient with recurrent ventricular tachyarrhythmia who underwent placement of an implantable cardioverter-defibrillator (ICD) and pituitary adenoma surgery. By searching in Medline (via PubMed), Embase and Lilacs, with the search strategies shown in Table 1, only one case report^7^ was found, similar to ours.

**Table 1.**
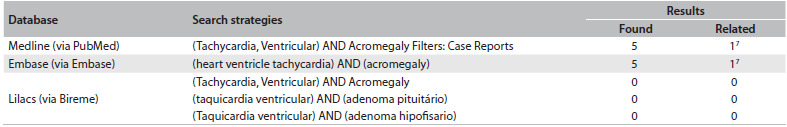
Search for case reports, in Medline (Medical Literature Analysis and Retrieval System Online), Excerpta Medica Database (Embase) and Lilacs (Literatura Latino-Americana e do Caribe em Ciências da Saúde) databases, performed on May 1, 2013

## CASE REPORT

**Figure 1.**
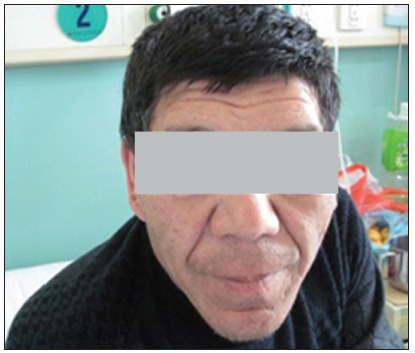
The patient exhibited the typical features of acromegaly, with increased head circumference and pronounced lower jaw protrusion.

A 50-year-old Han Chinese man was admitted to our hospital after several episodes of syncope during the previous week, with electrocardiographic evidence of monomorphic ventricular tachycardia. On physical examination, increased head circumference, pronounced lower jaw protrusion with macroglossia and swelling of the soft tissues in the hands and feet were evident (Figure 1).

Serum levels of growth hormone and serum insulin-like growth factor-1 (IGF-1) were elevated, reaching 40 and 368 ng/ml, respectively. The concentration of serum brain natriuretic peptide (BNP) was 2640 pg/ml. The patient’s medical history was remarkable because of the presence of systemic hypertension over the past five years, which had not been treated regularly by antihypertensive drugs. His daily blood pressure range was from 150/90 to 160/100 mmHg. Blood tests on admission revealed that the patient’s serum levels of estradiol, luteinizing hormone, prolactin and follicle-stimulating hormone were within normal values.

**Figure 2.**
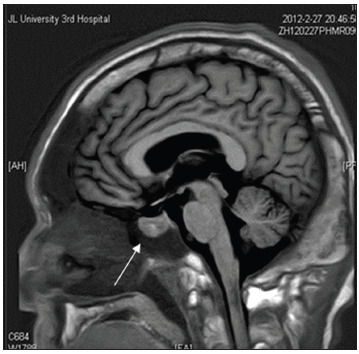
Findings from cerebral magnetic resonance imaging (MRI). An enlarged pituitary gland (arrowhead) appeared as a mass-like region with heterogeneous signal intensity and focal hyperintensity.

Cerebral magnetic resonance imaging (MRI) revealed an intrasellar mass lesion, suggestive of definitive presence of a pituitary adenoma (Figure 2). The size and shape of his skull were increased. Scattered spots and flaky hyperintensity in T1 and T2, with unclear edges, were observed in the basal ganglia bilaterally. The right corona radiata area and the fluid attenuated inversion recovers (FLAIR) images of these area showed medium intensity. Enlargement of the pituitary gland with uneven signal, patchy hyperintensity on T1 and hyperintensity on T2 was observed with a clear edge. The other brain parenchyma area did not show any abnormal signal. No obvious abnormalities were observed in the size and shape of the ventricles, sulci or cistern, and there was no schizencephaly. There was no midline structural shift, but part of the bifurcation of the left middle cerebral artery was slightly enlarged. Hyperintensity on T1 and T2 was observed in the maxillary sinus bilaterally, right ethmoid and frontal sinus. Positron emission tomography-computed tomography (PET-CT) fusion imaging did not reveal any malignant lymph node metastases.

The main echocardiographic values found through a transthoracic echocardiogram performed on this patient at the time of admission were as follows: left atrium (LA): 47.4 mm; left ventricle (LV): 71.8 mm; right ventricle (RV): 21.9 mm, ejection fraction (EF): 45%; fractional shortening (FS): 23%; interventricular septum thickness (IVS): 17.7 mm; and left ventricular posterior wall thickness (LVPW): 19.0 mm. Left ventricular hypertrophy, symmetry septal, left ventricular wall thickening, uncoordinated movements, diffuse wall motion abnormalities, moderate aortic regurgitation and mild mitral regurgitation were also found by means of echocardiography on this patient.

Coronary angiography showed normal coronary arteries. The patient received oral drug therapy consisting of aspirin, carvedilol and enalapril. At this time, a possible ICD insertion was planned for prevention of sudden cardiac death.

**Figure 3.**
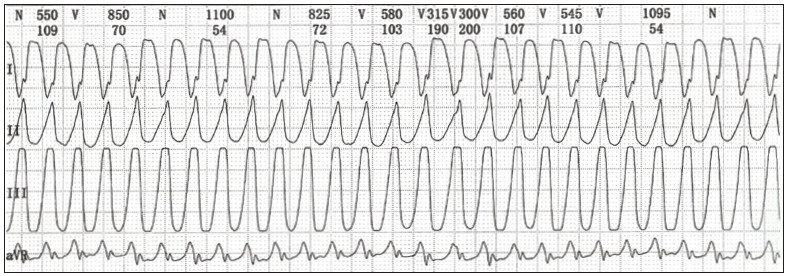
Findings from the electrophysiological test. Ventricular tachycardia found from electrocardiogram.

The patient was referred for electrophysiological study. Intermittent monomorphic ventricular tachycardia concomitant with syncope was induced by means of programmed electrical stimulation with three extrastimuli from two right ventricular sites, using a standard stimulation protocol (Figure 3). The implantation decision was supported by the 2006 guidelines of the American College of Cardiology/American Heart Association Task Force and the European Society of Cardiology Committee for management of patients with ventricular arrhythmias and prevention of sudden cardiac death.^8^

A dual-coil lead ICD defibrillator (Medtronic Marquis DR 7274) was implanted and a 50-mg dose of metoprolol succinate was administered twice per day. Transsphenoidal surgical resection of a pituitary adenoma was performed after the ICD implantation.

During six months of follow-up, no documented episodes of syncopal ventricular arrhythmia occurred.

## DISCUSSION

The risk of cardiovascular diseases is directly associated with elevated serum levels of growth hormone and prolonged clinical course. Chronic excessive growth hormone and IGF-1 secretion in acromegaly cases affects cardiac morphology and performance, thereby inducing cardiomyopathy specific to this disease.^9^^,^^10^ The early stage of acromegaly is characterized by hyperkinetic syndrome. Frequently, if the disease is untreated for many years or unsuccessfully treated at the early stage, concentric hypertrophy and diastolic dysfunction can develop. As the disease progresses to advanced stages, valve disease and impaired systolic and diastolic performance may occur. The pathological characteristics of acromegalic cardiomyopathy include interstitial fibrosis, myocyte apoptosis, and lymphocyte infiltration. The development of ventricular dysfunction and progression to cardiac failure may be related to increased myocyte apoptosis. The presence of myocardial fibrosis may be responsible for slowdown and non-homogeneity of propagation of action potentials,^11^^,^^12^ which induces ventricular arrhythmias.

Acromegalic patients often complain of various initial symptoms relating to cardiovascular or metabolic complications. Cardiovascular complications are common and account for as much as 60% of the mortality in acromegaly cases. Malignant ventricular tachyarrhythmias are commonly concomitant with recurrent syncope and are often a contributing factor in sudden cardiac death in acromegalic patients, even in those with an apparently normal heart.^13^ Therefore, when occurrences of clinical arrhythmias and heart failure are not easily explained by cardiogenic causes, it seems reasonable to consider the possibility of acromegaly-associated cardiovascular complications.

Hidden symptoms and slow clinical progression make acromegaly difficult to diagnose, particularly in the early stages. For this patient, ventricular tachyarrhythmia was definitively identified on admission, but its etiology was unclear. The possibility of acromegaly makes it extremely important to identify the specific heart disease responsible for arrhythmia when it occurs. In this case, echocardiographic examination did not meet all the diagnostic criteria for dilated cardiomyopathy, although dilated cardiomyopathy may cause arrhythmia.

After careful physical examination, the typical signs of acromegaly in this case were identified. Moreover, cerebral MRI revealed the presence of an intracranial pituitary adenoma, and blood tests indicated that the serum level of growth hormone was elevated.

Presence of a pituitary adenoma is responsible for excessive levels of growth hormone, which leads to acromegalic cardiomyopathy. Cardiovascular complications might benefit from treatment directed towards the pituitary adenoma, and lowering the blood growth hormone levels might arrest further progression of myocardial hypertrophy.^14^ Bromocriptine inhibits release and synthesis of prolactin by acting directly on the prolactin-secreting cells of the anterior pituitary.^15^ In patients with acromegaly, bromocriptine treatment results in reduction of serum growth hormone levels,^16^ which consequently blocks the unfavorable effect of growth hormone on the heart.

Several studies have found that early heart attack could be effectively controlled after successful treatment of pituitary tumors,^17^^,^^18^ thus indicating that early-stage cardiovascular complications are reversible. For example, recent evidence has shown that normalization of growth hormone and IGF-1 can work even in the presence of severe fibrosis in the myocardium.^7^ However, whether end-stage acromegalic cardiomyopathy is reversible is unknown. Unexpectedly, in the present case with end-stage acromegalic cardiomyopathy, after the resection of the pituitary adenoma, no arrhythmic episodes occurred during six months of follow-up.

## CONCLUSION

Malignant ventricular tachyarrhythmia might be effectively controlled by implantation of an ICD, surgical removal of a pituitary adenoma and oral drug therapy.
